# Serum Treatment of Bacillary Dysentery

**Published:** 1919

**Authors:** 


***Serum Treatment of Bacillary Dysentery.*** Abstract from the *Journal of the American Medical Association,* November 23, 1918.

According to Lantin the use of specific treatment seems to be an effective means of checking the progress of the disease in cases of true bacillary dysentery. Of twenty patients whom he had so treated only one patient died. Different methods of administering the serum, namely, intramuscularly, intravenously and by rectum (serum enema), have been employed. Out of seventeen cases in which cultures were made from the feces, six were negative. Of the eleven cases that were found to be positive, two were of the Shiga and one of the Flexner type. Of these twenty cases, five patients were treated medicinally, combined with intramuscular injection of serum, with one death ; six patients intramuscularly, with no deaths ; three patients treated both with intramuscular injections and anti-dysenteric serum by rectum, with no mortality ; three patients treated solely with serum by rectum, with no deaths ; and finally, three patients treated intravenously, with no deaths.

The serum by rectum was given in the following way. The patient was put in the knee chest position. The injection of the serum was preceded by a cleansing enema of 1.5 per cent. solution of sodium bicarbonate ; this was followed by another enema of starch solution with a few drops of tincture of opium (60 cc. with 10 drops of tincture of opium) to diminish the irritability of the intestine ; a half hour later the serum was given by rectum. The amount of serum used was from 30 to 50 cc. daily, depending on the severity of the case, although the serum can frequently be given without any danger and in larger doses. The intramuscular administration of serum was done with usual aseptic precautions. Twenty cc. of the serum were given twice a day, usually injected into the buttock. Larger doses may be given, depending, of course, on the severity of the case. Intravenous injection was done by the closed method, and under rigid asepsis into the median basilic vein. To avoid anaphylatic symptoms, 1 cc. of the serum may be injected intravenously about six hours before the full dose is given. Lantin's dosage was 10 cc. every other day.

